# Automated Skin Lesion and Cancer Detection Using Computer Vision: A Comprehensive Review

**DOI:** 10.3390/bioengineering13080872

**Published:** 2026-07-28

**Authors:** Bhagyashri S. Sonune, Udayakumar Ramanathan, Dhiraj P. Tulaskar, Shon G. Nemane, Madhusudan B. Kulkarni, Prakash Rewatkar, Manish Bhaiyya

**Affiliations:** 1 Department of Computer Science and Information Technology, Kalinga University, Raipur 492101, CG, India; 2 Department of Electronics and Telecommunication Engineering, Shri Sant Gajanan Maharaj College of Engineering, Shegaon 444203, MH, India; 3 Manipal Institute of Technology, Manipal Academy of Higher Education, Manipal, India; 4 Department of Mechanical Engineering, Israel Institute of Technology, Haifa 3200003, Israel

**Keywords:** skin lesion, cancer, computer vision, artificial intelligence (AI), detection, dermatology

## Abstract

Detection of skin cancer has become an increasingly prevalent health issue for which there is a need for reliable methods of detection to improve patient outcomes, minimize delays in diagnosis, and ensure clinical referral. Advances in computer vision and artificial intelligence have allowed automatic analyses of dermoscopic, clinical, and smartphone imaging of skin lesions. While numerous models have been found to perform very well on the basis of curated benchmark datasets, their accuracy in practical settings still needs to be evaluated. This review critically evaluates the literature from 2015 to 2025. Rather than considering the Dice, Jaccard, AUC, sensitivity, and specificity metrics on an absolute basis, this review evaluates them relative to dataset quality, validation process, external testing, statistical analysis, and risk of bias. Key areas of focus include dataset imbalance, lack of coverage of darker skin, poor external validation, explainability, uncertainty quantification, and barriers to clinical adoption. This review provides valuable insights for researchers, practitioners, dataset producers, and healthcare providers involved in developing AI-enabled dermatology tools.

## 1. Introduction

**Figure 1:**
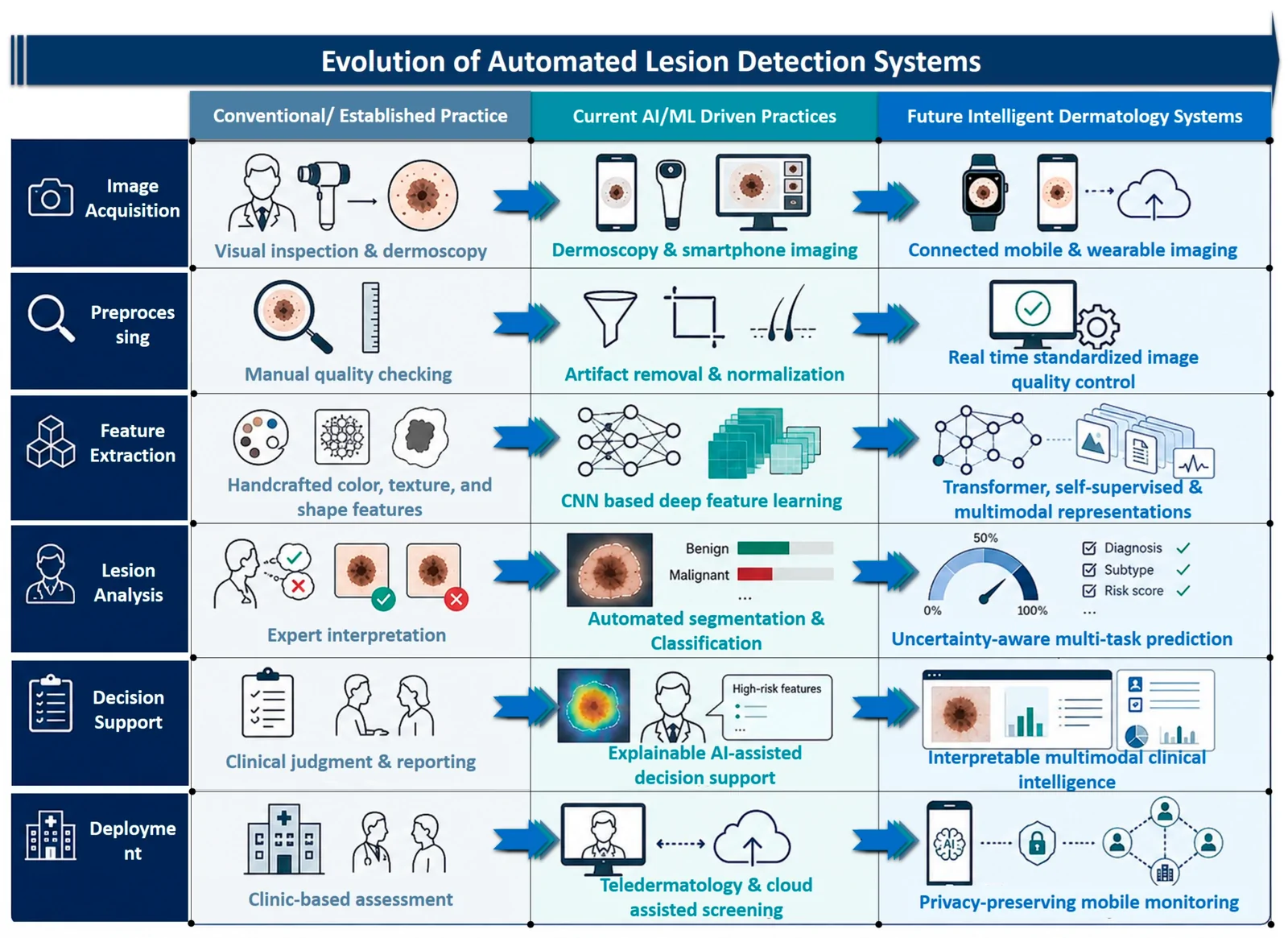
Evolution of skin lesion detection systems from conventional methods to AI-ML-empowered frameworks.

Skin cancer comprises one of the most prevalent and clinically significant types of cancer that exist in the world today, encompassing not only melanoma but also non-melanoma forms of skin cancer such as basal cell carcinoma and squamous cell carcinoma [1,2,3,4]. While the latter types may be more prevalent, the former, namely melanoma, poses great clinical concern due to its higher tendency for metastasis and associated risk of mortality when left undiagnosed [5,6,7,8]. The early identification of skin cancer is crucial not only for the improvement of patient survival but also for avoiding unnecessary biopsy procedures and for timely referral to specialists. Typically, the process of skin cancer diagnosis involves visual inspection and then dermoscopy, providing a better visibility of pigmentation structure, vasculature, boundaries of lesions, and other features that cannot be observed visually (see Figure 1). However, the effectiveness of the procedure will depend on many factors, such as the physician’s experience and level of training, quality of images, lesion morphology, anatomical site, patient skin pigmentation, and availability of dermoscopy or dermatology services [9,10,11].

There is a need for computer-aided diagnosis tools that will help eliminate subjectivity and provide objective information for the assessment of skin lesions. Previously, computer-aided diagnosis was based on engineered features such as asymmetry, border irregularity, pigmentation differences, diameter, texture, and shape characteristics [12,13,14,15]. Such methods were very useful in building a foundation for lesion characterization using computers; however, they were prone to various lighting effects, interference by hairs, image acquisition effects, segmentation errors, and low diversity of the datasets used. The fast development in machine learning and deep learning has dramatically changed the way skin lesions are assessed. There are many computer algorithms available that can analyze dermoscopic, clinical, and mobile phone pictures automatically using techniques such as convolutional neural networks, transfer learning, ensemble modeling, attention mechanisms, and transformers. Publicly available databases like ISIC, HAM10000, PH2, Derm7pt, and SD-198 have sped up the development of these algorithms through benchmarking [16,17,18,19,20,21].

However, despite all the progress, there are still problems with translating the use of AI-based algorithms into clinical practice. There are many reports about the good internal performance of such algorithms, where Dice and Jaccard scores for segmentation and AUC, accuracy, sensitivity, and specificity values for classification show promising results [22,23,24,25,26,27]. Nevertheless, these metrics are usually obtained using curated datasets, internal validation splits or image-level partitions that do not cover real-world clinical diversity. The differences in imaging devices used, acquisition protocol, lesion prevalence, annotation quality, class balance and demography could affect the performance of the models negatively when these algorithms are used on separate datasets [28,29]. Furthermore, the lack of representation of dark Fitzpatrick skin types, rare lesion sub-types, clinical images without dermoscopy and smartphone images is the problem of many public datasets [30,31,32,33].

Several review articles have summarized progress in skin cancer detection using machine learning, deep learning, convolutional neural networks, patient metadata, and clinical deployment perspectives. Earlier reviews mainly focused on conventional image-processing pipelines or CNN-based classification, while more recent systematic reviews have examined clinician–AI comparison, primary-care applicability, commercial dermatoscopic systems, Asian populations, and melanoma diagnosis or prognosis [34,35,36,37,38,39,40,41]. However, most previous reviews either emphasized classification alone, focused on dermoscopic images, discussed deep learning methods narratively, or did not integrate segmentation, classification, generative augmentation, multimodal fusion, explainable AI, fairness, external validation, and risk-of-bias appraisal within a single framework. Therefore, as summarized in Table 1, there remains a need for a more integrated and critically appraised review that evaluates not only what performance values are reported, but also how reliable those values are in terms of dataset quality, validation design, external testing, and clinical applicability.

### Novelties and Contributions of This Review

This review helps fill the abovementioned research gap through a systematic and critical orientation to synthesize skin lesion and cancer detection via computer vision and artificial intelligence approaches between 2015 and 2025. As opposed to prior reviews, which mainly considered classification accuracy, this review encompasses four related fields of study: lesion segmentation, image classification, data generation, and multimodal fusion. Furthermore, the review looks at emerging research trends, including transformer models, self-supervised learning, interpretable AI, uncertainty-aware prediction, and privacy-preserving federated learning. In total, 125 primary studies were reviewed from among 842 screened records through the PRISMA-oriented process detailed in the Methodology section.

The key contribution of this systematic review lies in the interpretation of Dice, Jaccard, AUC, sensitivity, and specificity scores in relation to the quality of the methodology as opposed to their direct comparability as outcome measures. In order to improve the systematic aspect of this manuscript, the review makes use of an appraisal perspective of PROBAST-AI/TRIPOD-AI type, taking into account representativeness of the dataset, quality of annotation, control of data leakage, validation approach, external validation, subgroup or fairness analysis, interpretability, reproducibility, and clinical significance.

This review is a consolidation of computer vision techniques used for the automatic detection of skin lesions and cancers. In addition to progress in the field, this review provides evidence of the challenges that still need to be overcome before such AI technology can be viewed as reliable and equitable.

## 2. Review Methodology

This review was conducted using a systematic and reproducible methodology designed to identify, screen, extract, and critically appraise studies on automated skin lesion and skin cancer detection using computer vision and artificial intelligence. The review process followed the PRISMA 2020 framework and included database searching, duplicate removal, title and abstract screening, full-text eligibility assessment, structured data extraction, and risk-of-bias/quality appraisal. In response to the methodological heterogeneity of artificial-intelligence-based diagnostic studies, the synthesis was not limited to reporting numerical performance values; instead, reported Dice, Jaccard, AUC, sensitivity, and specificity values were interpreted in relation to validation design, dataset characteristics, annotation quality, leakage control, and external validation.

### 2.1. Search Strategy and Information Sources

A comprehensive literature search was conducted across five major scientific databases: PubMed, IEEE Xplore, Scopus, Web of Science, and arXiv. The search covered studies published between 1 January 2015 and 31 March 2025. This time window was selected because the period after 2015 corresponds to the rapid expansion of deep-learning-based skin lesion analysis, particularly following the widespread adoption of convolutional neural networks, public dermoscopy benchmarks, and challenge datasets such as ISIC.

The search strategy combined lesion-related, imaging-related, and machine-learning-related terms. Representative search terms included: “skin lesion”, “skin cancer”, “melanoma”, “non-melanoma skin cancer”, “dermoscopy”, “clinical image”, “smartphone image”, “computer vision”, “artificial intelligence”, “machine learning”, “deep learning”, “convolutional neural network”, “CNN”, “transformer”, “segmentation”, “classification”, “GAN”, “generative model”, “multimodal fusion”, “metadata”, “explainable AI”, and “federated learning”. Boolean operators were used to combine these terms according to the syntax requirements of each database.

In addition to database searching, manual searches were performed using known benchmark resources and dataset-related literature, including the ISIC Archive, HAM10000, PH2, Derm7pt, and SD-198 datasets. The reference lists of eligible articles and relevant review papers were also screened to identify additional primary studies that may not have been captured through database searching.

### 2.2. Eligibility Criteria

Studies were included if they met the following criteria:

(i) They were primary research articles;
(ii) They addressed automated skin lesion or skin cancer analysis using dermoscopic, clinical, or smartphone-based images;
(iii) They investigated at least one of the following tasks: lesion segmentation, image classification, generative augmentation, multimodal fusion, explainability, or deployment-oriented AI methods;
(iv) They reported quantitative performance metrics such as THE Dice coefficient, Jaccard index, AUC, accuracy, sensitivity, specificity, precision, F1-score, or related diagnostic indicators.

Studies were excluded if they were review articles, editorials, commentaries, conference abstracts without sufficient methodological detail, non-image-based studies, non-skin-cancer studies, duplicate publications, or studies published before 2015. Studies using private datasets were excluded when the dataset origin, annotation protocol, class distribution, or evaluation design was insufficiently described. Studies that did not report quantitative outcomes or did not provide enough methodological information to assess model development and validation quality were also excluded.

### 2.3. Study Selection Process

The identification of all articles from the literature search was performed using a referencing procedure, during which any duplicate records were discarded. The next step of screening involved the removal of clearly irrelevant articles at the level of titles and abstracts by two independent reviewers. Finally, those potentially relevant articles that remained were independently assessed by two reviewers against predefined inclusion and exclusion criteria.

Methodological rigor was enhanced through inter-reviewer reliability testing via Cohen’s Kappa. Inter-reliability testing via Cohen’s Kappa produced high inter-reliability scores between the two reviewers for both screenings. The inter-reliability score via Cohen’s Kappa was 0.86 for the screening of titles and abstracts, while that of full texts was 0.82. Discrepancies were sorted out through discussions and if not settled, a third reviewer decided the issue.

**Figure 2:**
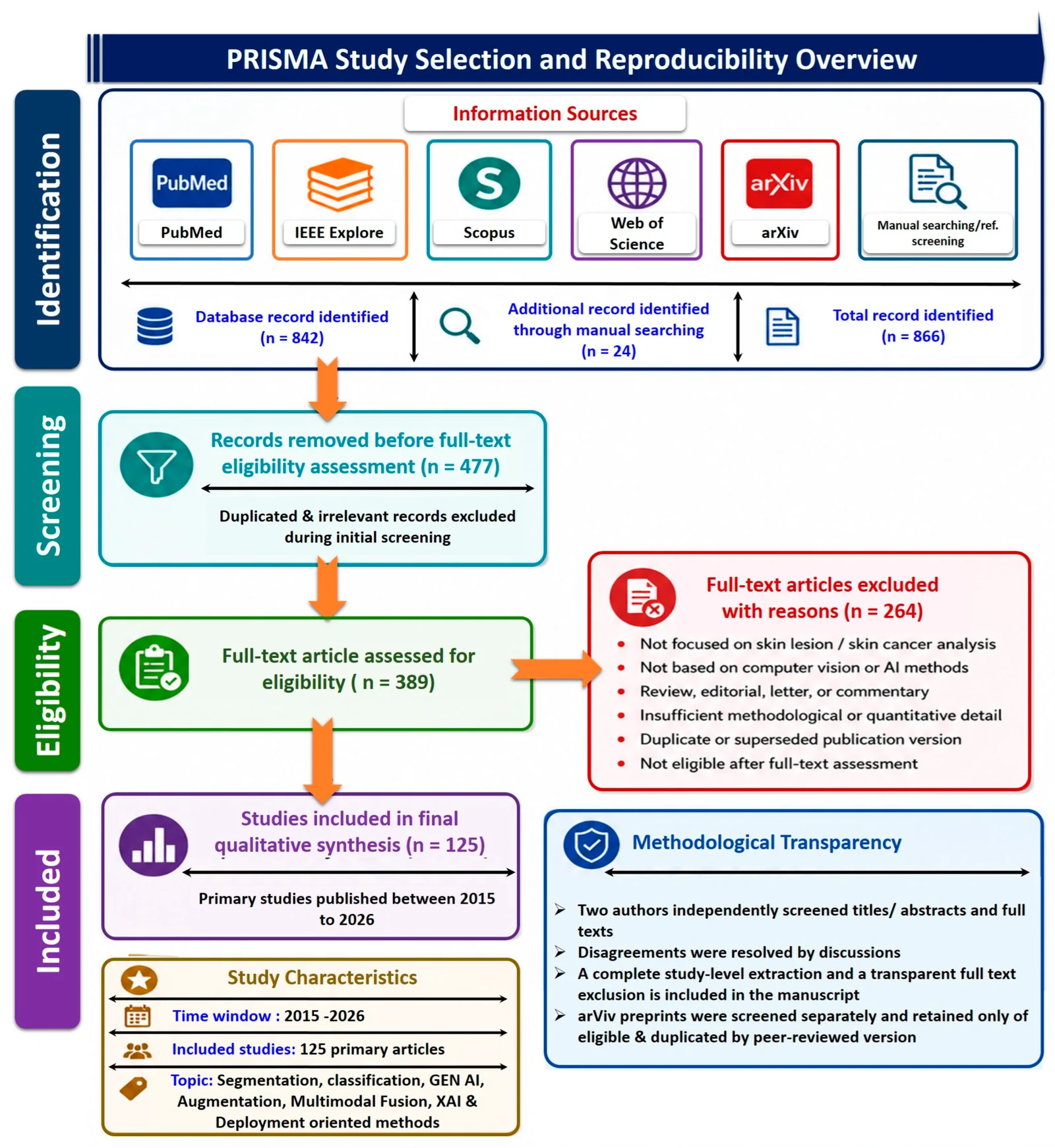
PRISMA 2020 study-selection and reproducibility workflow for the systematic review.

The first phase of the literature search generated 842 citations from searches through databases and other sources. After the elimination of duplicates and screening for eligibility, 125 primary studies were selected for inclusion in this systematic review. The selection of studies is illustrated by the PRISMA flow diagram in Figure 2 below. The studies were categorized based on their respective methodological approach as follows:

### 2.4. Data Extraction

Data were extracted using a standardized template to ensure consistency across studies. The extracted items included publication year, study objective, imaging modality, dataset name, dataset size, lesion classes, model architecture, preprocessing methods, augmentation strategy, segmentation or classification task, training–validation–test data split, validation approach, reported performance metrics, external validation status, interpretability method, and major limitations reported by the authors.

For segmentation studies, extracted metrics included the Dice coefficient, Jaccard index, sensitivity, specificity, boundary-related indicators, and annotation details where available. For classification studies, extracted metrics included AUC, accuracy, sensitivity, specificity, precision, F1-score, and class-wise performance where available. For generative augmentation studies, extracted information included GAN type, conditioning strategy, synthetic-image evaluation, effect on downstream classification or segmentation, and whether synthetic data improved external validation or only internal performance. For multimodal studies, extracted information included image features, clinical metadata, fusion strategy, missing-data handling, and performance comparison between image-only and multimodal models.

### 2.5. Quality Assessment and Risk-of-Bias Appraisal

To strengthen the systematic-review characteristic of this work, a formal quality-assessment and risk-of-bias appraisal was performed for the included AI diagnostic studies. The appraisal framework was adapted from PROBAST+AI and TRIPOD+AI-style principles. PROBAST+AI is intended for evaluating quality, risk of bias, and applicability of prediction models using regression or artificial intelligence methods, while TRIPOD+AI provides updated reporting guidance for prediction-model studies that use regression or machine-learning approaches.

Each study was assessed across the following domains: data source and population representativeness, reference standard or annotation quality, dataset splitting and leakage control, preprocessing transparency, augmentation strategy, model-development procedure, internal validation, external validation, performance reporting, fairness/subgroup analysis, interpretability, uncertainty estimation, reproducibility, and clinical applicability.

Each domain was rated as low concern, some concern, or great concern. An overall evidence-strength judgment was then assigned to each study based on the combined methodological profile. Studies with a transparent dataset composition, patient-level data splitting, reliable annotation or reference standards, complete performance reporting, and independent external validation were considered to provide stronger evidence. Studies based only on internal validation, single-dataset testing, unclear splitting strategy, incomplete metric reporting, or limited demographic diversity were considered to provide moderate or weaker evidence, even if their reported Dice, Jaccard, or AUC values were high.

This appraisal was used to avoid over-interpreting numerical performance metrics. For example, a segmentation model reporting a high Dice score on an internal ISIC test split was not interpreted as having the same strength of evidence as a model evaluated on an independent external dataset. Similarly, a classification model reporting high AUC without sensitivity, specificity, confidence intervals, calibration, or subgroup analysis was considered less clinically reliable than a model reporting complete diagnostic metrics and external validation. Supplementary Materials were added to improve transparency and reproducibility of the search, screening, extraction, and quality-assessment process. 

### 2.6. Appraisal Domains Used for Evidence Evaluation

Table 2 presents the quality assessment framework employed to evaluate the methodological quality and risk of bias of the included AI-based diagnostic studies.

### 2.7. Evidence-Strength Classification

Based on the risk-of-bias appraisal, the included studies were interpreted using three evidence-strength categories: *Stronger evidence*: These studies have clearly described datasets, reliable reference standards, patient-level splitting, complete metric reporting, and independent external or cross-dataset validation. These studies provide more reliable support for claims of model generalizability and clinical relevance.

*Moderate-quality evidence*: These studies have been developed in an openly accountable manner and validated internally, but not externally, or where the analysis of subgroups is partially complete, or where diagnostic criteria have only been partially reported.

*Poorer-quality papers*: These papers have ambiguous data partitioning, potential leakage, inadequate sample sizes or imbalance, lack of thorough reporting, lack of external validation, limited annotation information, and/or results obtained from internal test sets that have been overly curated. High Dice, Jaccard, AUC, sensitivity, or specificity results from such papers were not considered strong indicators of clinical readiness.

### 2.8. Data Synthesis

Because the included studies differed substantially in dataset composition, lesion categories, imaging modalities, preprocessing methods, model architectures, and validation protocols, a formal meta-analysis was not performed. Instead, the findings were synthesized qualitatively and comparatively. Performance values were summarized by methodological category, including segmentation, classification, generative augmentation, and multimodal fusion.

The reported Dice and Jaccard values for segmentation studies were interpreted together with annotation quality, lesion-boundary definition, and external-validation status. Similarly, AUC, sensitivity, and specificity values for classification studies were interpreted in relation to class imbalance, threshold selection, validation design, and dataset representativeness. For generative models, improvement in internal classification or segmentation performance was interpreted cautiously unless supported by independent testing. For multimodal studies, performance gains were evaluated in relation to metadata completeness, fusion strategy, and reproducibility.

Hence, the values provided for performance in this review cannot be seen as interchangeable across different studies. They are, rather, evidence that is shaped by the qualities of the dataset used, by the validation process, and by the risk of bias. In this way, the review can go further than just providing a narrative description of the evidence for AI performance in automated skin lesion and cancer detection.

## 3. Background, Clinical Context, and Dataset Characteristics

Skin cancers include both melanomas and non-melanoma skin cancers, like basal cell carcinoma and squamous cell carcinoma. Even though non-melanoma skin cancers are more prevalent than melanomas, the latter are clinically more aggressive due to their tendency to metastasize and high probability of fatalities in cases of late diagnosis [51,52,53]. Therefore, early detection is critical for increasing patients’ survival rates, minimizing unnecessary biopsies, and ensuring timely referrals to specialized dermatologists. As part of the usual clinical procedures, the initial step in the diagnostic process typically involves examination through the naked eye, with dermoscopy being employed when possible. Using dermoscopy allows an improved view to be obtained of the subcutaneous pigmentation network, vascular components, lesion margins, and morphology not visible through conventional examination of the skin. Yet, the efficacy of dermoscopy in skin cancer diagnosis greatly depends on a variety of factors [54,55,56].

A computer-aided diagnosis system has been designed in order to minimize diagnostic variance and ensure objectivity in skin lesion analysis. Previous computer-aided diagnosis systems were mostly based on hand-crafted features that included asymmetry, irregular borders, color heterogeneity, texture, size, and shape features [57,58,59,60]. This approach formed the basis of automated skin lesion analysis, but it was prone to changes in illumination, hair artifacts, noise, segmentation error, and hardware differences. The development of deep learning, especially the use of convolutional neural networks and transfer-learning approaches, led to changes in the field of automated skin lesion analysis from the manual engineering of features to automated feature learning [61,62,63,64].

The clinical value of AI-based skin lesion analysis depends strongly on the datasets used for training, validation, and testing. Public datasets such as ISIC, HAM10000, PH2, Derm7pt, BCN20000, PAD-UFES-20, SD-198, Fitzpatrick17k, and DDI have accelerated algorithm development by providing benchmark images for segmentation, classification, multimodal learning, and fairness evaluation. However, these datasets differ substantially in imaging modality, sample size, lesion categories, annotation quality, metadata availability, acquisition setting, and skin-tone representation. Therefore, performance values reported across studies should not be interpreted without considering dataset characteristics [65,66,67,68,69]. Table 3 summarizes representative datasets commonly used in automated skin lesion and skin cancer detection studies and highlights their strengths, applications, and limitations.

## 4. Computer-Vision Workflow for Automated Skin Lesion Analysis

**Figure 3:**
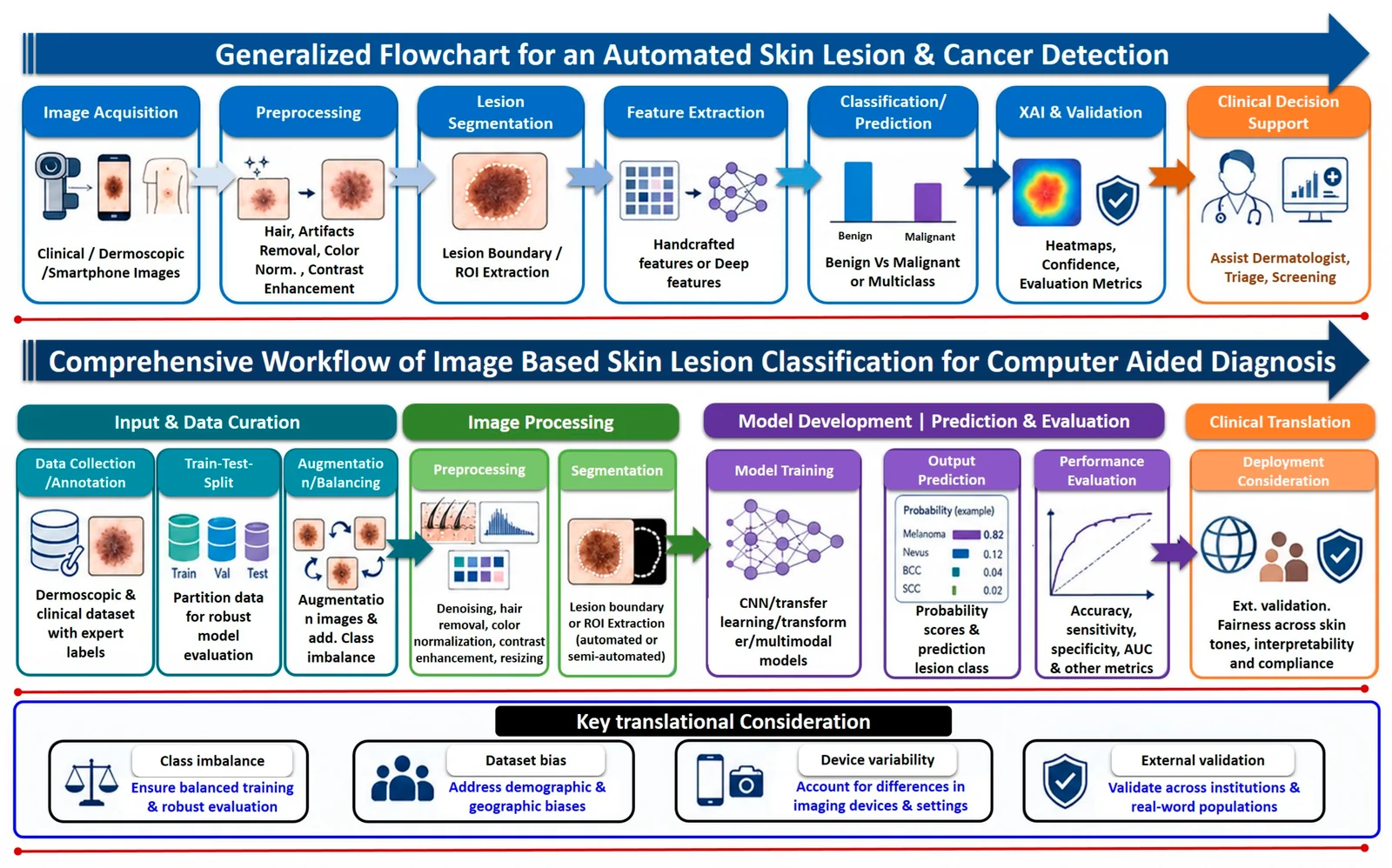
(**Top**) A Generalized flowchart for an automated skin lesion and cancer detection system and (**bottom**) a comprehensive workflow of image-based skin lesion classification for computer-aided diagnosis.

Automated skin lesion and skin cancer detection generally follows a sequential computer-vision workflow that converts raw dermatological images into clinically interpretable diagnostic outputs. This workflow connects image acquisition, preprocessing, lesion segmentation, feature extraction, classification, explainable AI, validation, and clinical decision support. As shown in Figure 3, the complete pipeline begins with dermoscopic, clinical, or smartphone-based image acquisition and proceeds through several computational stages before generating an output that can support dermatologist-assisted screening, triage, or diagnostic decision-making.

The first stage is image acquisition and data curation. Skin lesion images may be collected using dermatoscopes, clinical cameras, or smartphone devices. The quality of the acquired image strongly influences downstream model performance because dermatological images are sensitive to illumination variation, camera resolution, focus, acquisition angle, skin reflection, hair, ruler markings, and background skin texture. In addition to image collection, data curation includes diagnostic labeling, expert annotation, train–validation–test data splitting, and handling of class imbalance. Robust partitioning is essential because image-level random splitting or duplicate leakage may inflate reported model performance. Therefore, patient-level separation and careful dataset organization are required before model development.

The second phase is preprocessing, the goal of which is to enhance the image and reduce non-diagnostic artifacts. Typical preprocessing techniques include hair removal, normalization of colors, increasing the contrast of the image, scaling, cutting, reducing noise, and eliminating artifacts. Preprocessing is especially vital in skin lesions since the lesion image may differ significantly depending on the imaging equipment, lighting conditions, location of the lesion, and color of the skin. Nevertheless, preprocessing should be done with caution and transparency because it could distort the clinical structures of the lesion.

The next step involves lesion segmentation, where the boundaries of the lesion or the region of interest are defined. Segmentation helps to extract the lesion area from the rest of the normal skin around it for easier feature extraction and classification. Conventional approaches for segmentation involve thresholding, region growing, active contours, and morphological techniques, while modern approaches make use of U-Net, attention networks, residual networks, and a hybrid CNN–transformer architecture. Segmentation becomes critical when dealing with highly irregular lesions, lesions with low contrast and lesions with partial occlusion. Nevertheless, segmentation is heavily dependent on the quality of annotation, lesion boundaries, and expert validation masks.

Following segmentation, the process continues with feature extraction and modeling. Previous computer-aided diagnosis systems utilized features extracted manually in accordance with the asymmetry, borders, color, texture, and shape characteristics of lesions. Modern artificial intelligence systems rely on a deep neural network architecture for the automatic learning of hierarchical image features from lesion images. The use of CNNs, transfer learning, ensembles, attention mechanisms, and vision transformers is common at this stage. In more advanced AI systems, not only image-based features but also clinical metadata, such as age, gender, body part location, lesion history, and risk factors, can be incorporated into the diagnosis process [73,74,75,76].

Classification or prediction is the next phase. The type of classification done will depend on the purpose of the research. The model may either perform binary classification such as benign-versus-malignant prediction or multiclass classification involving melanoma, nevi, basal cell carcinoma, squamous cell carcinoma, seborrheic keratosis, dermo-fibroma, and other types of lesions. The output of models typically includes probabilities and diagnosis classes. Various measures used to evaluate the performance of the model include AUC, accuracy, sensitivity, specificity, precision, F1-measure, the Dice similarity coefficient, and Jaccard index. However, these metrics must be interpreted in relation to dataset composition, class imbalance, image modality, split strategy, skin-tone representation, and the external validation setting [46].

The last computational step concerns explainable AI and validation. The use of methods like Grad-CAM, saliency maps, attention maps, heatmaps, and confidence scoring can aid in understanding which parts of the image contributed to the model output. Such methods could increase physicians’ trust in models but cannot serve as proof of reliability in a clinical setting without validation with expert judgment and consideration of the lesion region. Validation should encompass not only internal testing but also cross-dataset testing and multi-institutional testing. External validation is especially crucial as a system that performs well on dermoscopic datasets might have different performance characteristics when tested on smartphone images, dark skin types, or rare lesion variants [38,77,78,79].

The clinical end point of this workflow is decision support. AI-driven solutions should help dermatologists, general practitioners, and tele-dermatology platforms in terms of screening, triaging, prioritizing referrals, lesion tracking, and risk stratification. They should not replace clinical expertise completely. An effective solution should not only provide the diagnosis but also estimate the level of confidence of the prediction, estimate uncertainty, generate an explanation map, and suggest referring a case to an expert in a situation of uncertainty and high-risk predictions. This is why the presented workflow in Figure 3 reveals the necessity of integrating technical and clinical aspects of AI model development and deployment.

## 5. AI-Based Methodological Approaches for Automated Skin Lesion Analysis

From the computer vision workflow discussed in Section 4, we can deduce that skin lesion analysis using automation does not constitute an isolated step but rather a chain that consists of image capture, image preprocessing, skin lesion localization, feature extraction, classification, explanation, validation, and clinical decision-making. In this workflow, segmentation, classification, generation, and multimodal approaches have complementary functions. Segmentation localizes the lesion area and minimizes the effect of the normal skin around the lesions or any image artifacts. Classification maps the image features into diagnostic decisions such as benign or malignant lesions. Generative approaches handle the problems of unbalanced classes and limited data by generating synthetic or augmented skin lesion images, while multimodal approaches integrate image-based and clinical metadata information.

Consequently, the sections that follow present the different methodologies in automated skin lesion and cancer detection. Rather than viewing the Dice, Jaccard, AUC, accuracy, sensitivity, and specificity scores provided by researchers as comparable across different studies, the following discussion explains these measures in the context of the dataset used, validation procedure, external evaluation, variance/uncertainty reporting, and dataset heterogeneity. The organization of this discussion bridges the workflow explained in Section 4 and the improved comparison tables presented below.

### 5.1. Segmentation Methods

Lesion segmentation forms the basis of automatic skin lesion analysis since it defines the region of interest that contains the features used in diagnostics. Accurate segmentation enables the distinction between the lesion and normal skin tissue, hair, ruler lines, illumination variations, and other background artifacts. This becomes especially crucial for cases where the lesion is irregular, has low contrast, is heterogeneous, or is partially hidden. For conventional computer-assisted diagnostic systems, lesion segmentation was usually accomplished through techniques such as thresholding, regional growth, active contours, edge detection, and morphology. Despite being computationally easy, they were very prone to variations due to illumination conditions, lesion color variations, presence of artifacts, and manually set parameters.

The problem of detecting lesion boundaries using deep-learning-based segmentation techniques was significantly enhanced. Encoder–decoder-based networks, such as the U-Net architecture, along with its variations, gained popularity due to their ability to incorporate the task of feature extraction as well as spatial localization by making use of skip connections. Attention mechanism, residual layers, feature aggregation at multiple scales, adversarial training, and hybrid CNN–transformers were some of the more recent improvements for better localization of the boundary with a broader context [80,81,82,83,84,85].

However, segmentation performance is strongly affected by annotation quality and validation design. Dice and Jaccard scores depend not only on model quality but also on how lesion masks are generated. A polygonal expert mask, a rough manual outline, and a consensus annotation may produce different performance estimates. Similarly, internal testing on ISIC or PH2 does not necessarily indicate that the model will generalize to clinical or smartphone images. For this reason, segmentation results should be interpreted together with dataset type, annotation protocol, cross-dataset validation, and external testing status. A traceability-enhanced comparison of representative segmentation studies is presented in Table 4, which reports the method, dataset, validation design, extracted metrics, availability of confidence/variance measures, external-validation status, and dataset-level limitations.

Overall, segmentation methods have evolved from rule-based boundary detection to deep learning and attention-guided architectures. Nevertheless, their clinical reliability remains limited by inconsistent annotation standards, lack of external validation, and limited testing on diverse skin tones and imaging devices. Future segmentation studies should report mask-generation protocols, inter-annotator variability, patient-level data splitting, confidence intervals, and cross-dataset performance.

### 5.2. Classification Methods

Classification is the central diagnostic stage of automated skin lesion analysis. After image preprocessing and, in many pipelines, lesion segmentation, classification models assign diagnostic labels or risk scores to the lesion. The task may be binary, such as benign versus malignant classification, or multiclass, involving melanoma, nevus, basal cell carcinoma, squamous cell carcinoma, actinic keratosis, seborrheic keratosis, dermatofibroma, vascular lesions, and other lesion categories. Classification models are clinically important because their outputs can support screening, triage, referral prioritization, and dermatologist decision support.

In previous systems, features derived from dermatological guidelines, such as asymmetry, border irregularity, color variance, diameter, texture, and shape attributes, were manually crafted. These features were typically coupled with classic machine learning algorithms like support vector machine, K-nearest neighbor, decision tree, random forest, and artificial neural network. Though valuable in the past, manual feature selection techniques had poor performance in the presence of variability in illumination, imaging devices, skin color, lesion appearance, and segmentation [91,92,93].

The adoption of CNN revolutionized classification from manually designed feature extraction techniques to feature extraction through end-to-end learning. The following CNN-based models were used to perform classification tasks on several image datasets such as ISIC, HAM10000; that is, the Inception model, ResNet, DenseNet, VGG, EfficientNet, and MobileNet, among others. Transfer learning has gained popularity due to the unavailability of annotations for medical images compared with natural images. Transformer models and CNN–transformer hybrid models have also been tested for classification due to their ability to capture long-range dependency and global information in the image [48,64].

Even with good AUC and accuracy, classification results need to be carefully considered. Many studies apply internal validation, and even random splits by images may lead to leakage of information. The problem of class imbalance is also significant because of an excessive number of benign lesions and the low frequency of melanoma and other rare skin cancer types. In this way, accuracy could be very high despite low sensitivity for malignant classes. Thus, in addition to accuracy and AUC, classification studies have to include data about sensitivity, specificity, confidence intervals, metrics per class, calibration, external validation, and analysis of subgroups according to the Fitzpatrick skin type. Table 5 presents selected examples of classification studies with a focus on the model architecture, dataset, validation method, metric traceability, comparison of statistics, and limitations of datasets.

### 5.3. Generative Models and Multimodal Approaches

Generative modeling and multimodal learning are two of the essential improvements made to the segment/classify workflow. They help solve two major problems in skin lesion AI detection: the shortage of annotated data and lack of clinical context. The publicly available skin datasets are not balanced enough since there is limited content on malignant lesions, few rare cases, and low coverage of dark skin. Generative models try to improve the data balance by generating additional images or improving masks for lesions. Multimodal models are trying to make the AI systems more clinically realistic by integrating the images of lesions together with patient data [98,99].

Generative adversarial networks have been applied in the synthesis of dermoscopy images, in the refinement of segmentation masks, in balancing class weights and in generating underrepresented classes of lesions. In conditional GANs, one can generate images using class labels or segmentation masks, whereas some of the other GAN applications target high-resolution generation of lesion images or improvement of the segmentation process. While such applications might help improve the performance of classification and segmentation within the dataset, the generated synthetic images might contain unrealistic textures or repeating patterns that are not clinically meaningful. Learning such synthetic features can lead the classifier to perform very well internally but poorly during external validation. Hence, such models need to be evaluated by their performance, as well as by external validation, fidelity, diversity, and dermatologist evaluation metrics [90,100,101,102].

Another relevant area is multimodal learning, since dermatologists do not make diagnoses based on image data only. Clinical diagnosis includes age, localization, gender, risks, symptoms, progression of lesions, and dermoscopy criteria. Multimodal AI systems combine image features with metadata using early fusion, late fusion, attention-based fusion, or transformer-based fusion. Studies using datasets such as Derm7pt and PAD-UFES-20 suggest that metadata can improve performance compared with image-only models, especially when lesion images alone are ambiguous. However, multimodal learning introduces new challenges, including missing metadata, inconsistent clinical variables, demographic bias, and reduced reproducibility across datasets [71].

Generative and multimodal studies should therefore be interpreted in relation to their validation design and evidence strength. A small improvement in internal AUC after GAN augmentation does not necessarily prove clinical usefulness. Similarly, a multimodal model may perform well on one dataset but fail when metadata fields are missing or collected differently in another institution. Table 6 summarizes representative generative augmentation and multimodal studies, including their approach, dataset or modality, reported impact, availability of statistical information, key heterogeneity concerns, and evidence interpretation.

## 6. Challenges, Limitations, and Future Directions

Although automated skin lesion and skin cancer detection systems have achieved considerable progress during the last decade, several methodological, clinical, and deployment-related limitations continue to restrict their reliable translation into real-world dermatology practice. Many studies report high Dice, Jaccard, AUC, sensitivity, specificity, and accuracy values on curated public datasets; however, these values are often obtained under internal validation settings and may not fully reflect performance across different populations, institutions, imaging devices, skin tones, lesion sub-types, and clinical workflows. Therefore, future development should shift from isolated benchmark optimization toward clinically robust, externally validated, fair, interpretable, and workflow-compatible AI systems.

### 6.1. Dataset Bias and Benchmark Design

Among some of the main drawbacks of current research on skin lesions using AI techniques is the use of a small number of open-source datasets like ISIC, HAM10000, PH2, Derm7pt, and SD-198. While these have helped push forward the development of computer-based lesion detection methods, there are some constraints associated with their use. This includes factors like datasets having class imbalances, the presence of too many benign lesions, few cases of rare types of cancerous lesions, variable quality of labeling, and a lack of complete clinical data. Moreover, dark skin colors have been underrepresented in these datasets [107].

Future benchmark datasets need to have more diversity from both clinical and demographic perspectives. This will involve incorporating details such as lesion classification, histopathologic confirmation, lesion location, age, gender, scanning modality, setting, and Fitzpatrick skin type. Future benchmark protocols should involve patient-based data splitting, exclusion of duplicate images, fixed training, validation, and test sets, as well as a clear preprocessing pipeline. Reporting the results of future benchmarks needs to move away from aggregated area under the curve and Dice score reports and embrace individual subgroup analysis, calibration, and confidence interval reporting [23,108,109,110].

### 6.2. External and Multi-Institutional Validation

The first important issue contributing to the overestimation of performance in studies on the detection of skin lesions using AI is that, most often, researchers apply an internal data split based on the same dataset. The use of the same source of data for both training and testing can lead to a model that performs well only with images taken in the same setting, using the same device and with the same patients.

Future work must emphasize external validation with datasets from other institutions and geographic locations. Cross-dataset validation must become mandatory in order for claims of generalization to be made. Multi-institution validation would help better assess the performance of models in handling differences in image resolution, illumination, appearance of the lesion, camera use, dermoscopy technique, and patient characteristics. External validation in a prospective clinical setting would help ascertain if the use of artificial intelligence would help improve diagnostic decision-making, referral prioritization, screening efficiency, and patient outcomes [111,112,113,114].

### 6.3. Fairness Across Fitzpatrick Skin Types

The diversity of skin tones is one of the main limitations of existing studies on dermatology. Many freely accessible databases have a great proportion of images related to lighter skin tones, especially Fitzpatrick skin types I-III. Therefore, models based on such databases might have a lower efficiency in terms of darker skin tones, leading to potential delays in the detection of lesions among underrepresented groups. This limitation is vital due to the difference in the manifestation of malignant lesions on different skin tones and anatomical locations.

Performance of the model across all Fitzpatrick skin types I-VI should be reported by future researchers if metadata is available. Fairness assessment should include sensitivity, specificity, false-positive and false-negative rates, AUC, and calibration errors for specific subgroups. Datasets should be created to include darker skin tones, rare lesion appearances, acral and mucosal lesions, and anatomically diverse image samples. Additionally, the development of models should consider the effect of domain adaptation, reweighing, balanced sampling, synthetic augmentation, and fairness-aware learning [23,72,113,115].

### 6.4. Interpretability and Explainable AI

Explainable AI has become increasingly important in automated skin lesion diagnosis because clinicians need to understand whether a model is focusing on clinically meaningful lesion structures or on irrelevant image artifacts. Commonly used explanation methods include Grad-CAM, saliency maps, Integrated Gradients, occlusion sensitivity, attention visualization, and perturbation-based approaches. These methods generate visual maps that highlight image regions contributing to the model prediction. In skin lesion analysis, reliable explanations should ideally correspond to lesion borders, pigment networks, asymmetry, color variegation, vascular structures, ulceration, or other clinically meaningful dermoscopic patterns [116].

However, the reliability of explainable AI methods remains limited. Saliency maps are often noisy and may change substantially with small image perturbations. Grad-CAM can provide more visually interpretable heatmaps but may highlight broad regions rather than precise lesion structures. Integrated Gradients offers pixel-level attribution but depends on baseline-image selection and may be difficult for clinicians to interpret directly. Attention maps from transformer-based models are sometimes interpreted as explanations, but attention weights do not always correspond to causal diagnostic reasoning. Therefore, explanation outputs should not be treated as proof that a model is clinically reliable [38,117].

One key issue is that AI models might learn shortcuts from features not related to lesions, like rulers, marks of ink, hair, edges, color calibration markers, acquisition artifacts, or background skin structure. This could result in high internal accuracy, yet decrease external generalization. Visual explanations may look very convincing even in the case where there are spurious correlations. It means that visual analysis of heatmaps is not enough. Evaluation of explanation quality should be done quantitatively based on the degree of localization overlap with expert lesion masks, deletion–insertion tests, perturbation robustness, pointing game performance, clinically meaningful region detection, and diagnostic structure alignment.

From a clinician’s point of view, explanations should increase trustworthiness, help error detection, and aid decision-making, but not generate nice-looking heatmaps. Clinicians need explanations that are stable, interpretable, and dermatologically motivated. Therefore, future studies should involve not only technical metrics but also clinical evaluations of explainable AI. Future research should consider whether explanations help clinicians detect model errors, increase their diagnostic confidence, avoid false reassurance, and make decisions about referrals. Overall, explainable AI is a necessary but not sufficient aspect of clinical translation [118,119].

### 6.5. Uncertainty Estimation and Risk-Aware Decision Support

However, most modern AI solutions provide either a class label or a probabilistic output, without giving information about the reliability of that prediction. This is an issue in the medical field because AI might give very reliable-looking outputs based on low-quality images, uncommon types of lesions, out-of-distribution instances, or images from new types of devices. A clinically valuable solution must be able to recognize the cases where it is uncertain and advise on getting an expert opinion instead of providing a possibly unreliable prediction.

Future medical AI solutions need to include uncertainty assessment and risk-based decision support. Methods like MC-dropout, deep ensembles, Bayesian neural networks, temperature scaling, conformal prediction, and out-of-distribution detection can aid in assessing the confidence of a prediction. Rather than simply giving a binary output as to whether a lesion is benign or malignant, the model needs to provide risk values, uncertainty estimates, and recommendations on whether the case should be referred to a dermatologist [120,121,122].

### 6.6. Deployment Constraints and Clinical Workflow Integration

The other major limitation is the fact that most of the studies in skin-lesion AI are conducted offline through retrospective datasets. However, deployment in practice requires more than benchmark accuracies achieve. For successful clinical integration of the system, issues such as inference speed, hardware requirements, image quality management, data privacy, user interface design, clinician adoption, medicolegal liabilities, regulation, and integration into EHR or Teledermatology need to be addressed.

Future studies need to focus on evaluating the AI system within the context of the clinical workflow. It needs to be clear that the use of AI will be to support decision-making and not to replace dermatologists. Evaluation of models would involve how well AI can help dermatologists, general practitioners, and Teledermatologists in patient triaging, referral prioritization, monitoring of lesions, and second opinions. Evaluation of the deployment should consider not just the diagnostic accuracy but also issues related to workflow optimization, efficiency, timeliness, clinician confidence, risk of false reassurance, patient safety, and usability [123,124,125]. Table 7 summarizes the major limitations and corresponding future recommendations for developing clinically reliable skin-lesion AI systems.

## 7. Conclusions

The present review offers a comprehensive, critical analysis of studies of automatic skin lesion and cancer recognition through computer vision and artificial intelligence methods. Overall, deep learning demonstrated great promise for lesion segmentation, image classification, generative data augmentation, and multimodal decision support. Nevertheless, highly cited Dice, Jaccard, AUC, sensitivity, and specificity measures need to be understood carefully due to the presence of internal validity assessment, imbalanced datasets, underrepresentation of skin tones, and lack of confidence intervals reported and external validation in many studies. Through the combination of methodological comparison, risk-of-bias assessment, heterogeneity of datasets, explainability, fairness, and deployability, this review demonstrates that beyond benchmarking accuracy, there is much more to clinical translation. Future progress in this field will require multi-institutional, heterogeneous datasets, patient-level validation, uncertainty-based approaches, and clinically viable AI systems.

## Figures and Tables

**Table 1:** Comparative analysis of existing review articles and novelty of the present.

Published Review	Main Focus	Key Contribution	Limitation/Gap	How the Present Review Is Different
Mehta and Shah, 2016 [42]	Computer-aided skin cancer diagnosis	Summarized classical CAD steps such as preprocessing, segmentation, feature extraction, and classification.	Mainly focused on traditional image-processing pipelines before the deep-learning expansion.	Extends beyond classical CAD to CNNs, transformers, GANs, multimodal fusion, explainability, and clinical translation.
Adegun and Viriri, 2021 [4]	Deep learning for skin lesion analysis and melanoma detection	Provided a survey of deep-learning techniques used for skin lesion images.	Primarily technique-oriented; limited formal evidence-strength appraisal.	Adds critical appraisal of reported metrics using validation quality, external testing, and risk-of-bias considerations.
Dildar et al., 2021 [43]	Deep learning for skin cancer detection	Reviewed deep-learning-based methods for early diagnosis of skin cancer.	Strong classification emphasis; limited integration of multimodal and generative methods.	Integrates segmentation, classification, GAN-based augmentation, multimodal learning, and deployment challenges.
Höhn et al., 2021 [44]	CNNs with patient data	Systematically reviewed integration of patient metadata into CNN-based skin cancer classification.	Focused specifically on patient-data integration.	Discusses multimodal fusion as one component within a broader AI pipeline including segmentation, classification, and generative models.
Haggenmüller et al., 2021 [35]	CNN classification compared with human experts	Reviewed CNN-based skin cancer classification studies involving human experts.	Mainly clinician-comparison oriented.	Places classification performance within broader concerns of dataset bias, external validation, fairness, and interpretability.
Jones et al., 2022 [45]	AI/ML for early skin cancer detection in primary and community care	Evaluated AI/ML algorithms for early diagnosis in community and primary-care settings.	Strong clinical-setting focus but less emphasis on technical evolution across segmentation, GANs, transformers, and multimodal AI.	Connects technical model development with real-world clinical-readiness barriers.
Wu et al., 2022 [34]	Skin cancer classification with deep learning	Reviewed recent developments in dermoscopic skin lesion classification.	Mostly classification-centered and dermoscopy-focused.	Expands scope to segmentation, generative augmentation, multimodal fusion, explainability, and evidence strength.
Debelee et al., 2023 [46]	Skin lesion classification, segmentation, and detection	Surveyed recent machine-learning methods for skin lesion analysis.	Broad method summary with limited formal risk-of-bias interpretation.	Adds structured quality appraisal and interprets metrics according to validation rigor.
Nazari and Garcia, 2023 [47]	Skin cancer detection using clinical images	Focused on standard clinical images rather than only dermoscopy.	Does not comprehensively cover dermoscopic benchmarks, generative models, and multimodal fusion.	Combines dermoscopic, clinical, and smartphone-image perspectives with AI-model comparison.
Salinas et al., 2024 [48]	AI versus clinicians for skin cancer diagnosis	Systematic review and meta-analysis comparing AI and clinician performance.	Focused mainly on diagnostic accuracy comparison.	Goes beyond AI-versus-human comparison to assess methodological reliability, bias, and deployment readiness.
Ang et al., 2025 [49]	AI skin cancer detection in Asian populations	Addressed population-specific concerns and adaptation of AI models to Asian cohorts.	Region/population-specific scope.	Includes fairness and subgroup limitations as part of a broader global review framework.
Karimzadhagh et al., 2026 [50]	Umbrella review of systematic reviews and meta-analyses	Synthesized evidence from prior reviews and meta-analyses.	Umbrella-level focus; less detailed technical synthesis of segmentation, GANs, transformers, and multimodal methods.	Provides method-level synthesis with detailed discussion of AI pipelines, metrics, validation, and clinical translation.

**Table 2:** Quality-assessment framework used for included AI diagnostic studies.

Domain	Key Items Assessed	Relevance to Evidence Strength
Data source and representativeness	Dataset size, lesion diversity, class balance, imaging source, skin-tone diversity, single-center or multi-center origin	Determines whether the reported model performance is likely to generalize beyond the training dataset
Reference standard/annotation quality	Histopathology, expert diagnosis, consensus labels, segmentation-mask quality, annotation protocol	Weak labels or inconsistent masks can distort Dice, Jaccard, sensitivity, and specificity
Dataset splitting and leakage control	Patient-level separation, duplicate removal, train–validation–test data split, cross-validation strategy	Image-level splitting or duplicate leakage may inflate performance metrics
Preprocessing transparency	Hair removal, resizing, color normalization, artifact removal, contrast enhancement	Poorly described preprocessing reduces reproducibility
Augmentation strategy	Conventional augmentation, oversampling, GAN-based synthesis, minority-class balancing	Augmentation can improve internal metrics but may not improve external generalization
Model-development strategy	Architecture, transfer learning, hyperparameter tuning, ensemble method, threshold selection	Excessive tuning on benchmark datasets may overestimate real-world performance
Validation design	Internal validation, cross-dataset validation, external validation, multi-site testing	External validation provides stronger evidence than single-dataset internal testing
Performance reporting	Dice, Jaccard, AUC, accuracy, sensitivity, specificity, confidence intervals, calibration	Complete reporting allows fair comparison across studies
Fairness and subgroup analysis	Fitzpatrick skin type, age, sex, anatomical site, rare lesion classes	Lack of subgroup analysis limits clinical reliability and equity
Interpretability and uncertainty	Grad-CAM, saliency maps, uncertainty estimation, calibration curves	Supports clinical trust but does not replace external validation
Reproducibility	Public code, model details, dataset availability, parameter reporting	Determines whether results can be independently verified
Clinical applicability	Inference time, computational cost, workflow integration, deployment setting	High benchmark performance may not translate to practical clinical use

**Table 3:** Representative datasets commonly used in automated skin lesion and skin cancer detection [65,67,70,71,72].

Dataset	Image Modality	Dataset Size/Classes	Annotation and Clinical Information	Common Use in AI Studies	Strengths	Key Limitations
**ISIC Challenge/ISIC Archive**	Mainly dermoscopic images	ISIC 2019 includes **25,331 images** across **nine diagnostic categories**	Diagnostic labels; segmentation masks available in selected challenge tasks	Segmentation, classification, benchmarking, challenge-based comparison	Large international benchmark; widely used for model comparison	Mostly dermoscopic; class imbalance; performance may not generalize to clinical or smartphone images; external validation remains necessary (ISIC Challenge https://challenge.isic-archive.com/landing/2019/?utm_source=chatgpt.com)
**HAM10000**	Dermoscopic images	**10,015 dermoscopic images** across common pigmented lesion categories	Diagnostic labels; more than half of lesions confirmed by pathology, with others confirmed by follow-up, expert consensus, or confocal microscopy	Multiclass classification, transfer learning, benchmarking	Large, widely used, multi-source dermoscopic dataset	Class imbalance; limited skin-tone diversity reporting; high internal performance may be dataset-specific (PMC https://pmc.ncbi.nlm.nih.gov/articles/PMC6091241/?utm_source=chatgpt.com)
**PH2**	Dermoscopic images	**200 images**: 80 common nevi, 80 atypical nevi, and 40 melanomas	Lesion masks and diagnostic information	Segmentation validation and small-scale classification	Expert masks and well-defined lesion categories	Small sample size; limited lesion diversity; unsuitable alone for training large deep-learning models (PMC https://pmc.ncbi.nlm.nih.gov/articles/PMC8046537/?utm_source=chatgpt.com)
**Derm7pt**	Dermoscopic and clinical lesion images with metadata	**1011 lesion cases**	Seven-point checklist criteria, diagnosis, and metadata	Multimodal learning, checklist prediction, clinically interpretable AI	Useful for linking image-based AI with clinically meaningful dermoscopic criteria	Moderate size; missing or variable metadata may affect multimodal reproducibility (ResearchGate https://www.researchgate.net/publication/324381190_7-Point_Checklist_and_Skin_Lesion_Classification_using_Multi-Task_Multi-Modal_Neural_Nets?utm_source=chatgpt.com)
**BCN20000**	Dermoscopic images	Approximately **18,946–19,424 dermoscopic images** collected at Hospital Clínic Barcelona	Diagnostic labels; includes challenging lesion presentations	Large-scale classification and ISIC-related benchmarking	Includes difficult real-world cases such as nail/mucosal lesions, large lesions, and hypopigmented lesions	Single-institution origin; dermoscopy-focused; requires external validation across other centers (PMC https://pmc.ncbi.nlm.nih.gov/articles/PMC11183228/?utm_source=chatgpt.com)
**PAD-UFES-20**	Clinical images, mostly smartphone-acquired	**2298 samples** across six/seven lesion categories depending on grouping	Clinical images with patient metadata such as age, lesion location, Fitzpatrick skin type, and lesion diameter	Clinical-image classification, smartphone-based diagnosis, multimodal learning	Real-world clinical-image dataset with patient-level metadata	Smaller than ISIC/HAM10000; geographically specific; acquisition-device variability may affect generalization (PMC https://pmc.ncbi.nlm.nih.gov/articles/PMC7479321/?utm_source=chatgpt.com)
**SD-198**	Clinical/macroscopic images	**6584 images** across **198 skin disease categories**	Disease-category labels	Broad skin-disease classification and transfer-learning studies	Covers many dermatological conditions beyond melanoma	Not skin-cancer-specific; many categories have limited images; label and class heterogeneity affect benchmarking
**MED-NODE**	Clinical/non-dermoscopic images	Commonly reported as **170 images**: 70 melanoma and 100 nevi	Diagnostic labels	Binary melanoma/nevus classification using clinical images	Useful for non-dermoscopic evaluation	Very small dataset; limited diversity; not sufficient for standalone deep-learning training (GitHub https://github.com/openmedlab/Awesome-Medical-Dataset/blob/main/resources/MED-NODE.md?utm_source=chatgpt.com)
**Fitzpatrick17k**	Clinical dermatology images	**16,577 clinical images** with skin condition and Fitzpatrick skin-type labels	Diagnosis labels and Fitzpatrick skin-type annotations	Fairness analysis, skin-tone bias evaluation, clinical-image classification	Important dataset for evaluating skin-type-related performance disparities	Not limited to skin cancer; class imbalance and skin-type imbalance remain concerns (GitHub https://github.com/mattgroh/fitzpatrick17k?utm_source=chatgpt.com)
**DDI: Diverse Dermatology Images**	Clinical images	**656 images** from diverse skin tones and uncommon diseases	Expert-curated and pathologically confirmed images	Fairness testing and external validation of dermatology AI	Valuable for evaluating model performance across diverse skin tones	Small dataset; mainly useful for fairness testing and external validation rather than large-scale training (arXiv https://arxiv.org/abs/2203.08807?utm_source=chatgpt.com)

All websites are accessed on 23 June 2019.

**Table 4:** Comparison of representative skin lesion segmentation studies.

Study/Link	Method	Dataset/Image Type	Validation Design	Reported Metrics	CI/SD/Variance	External Validation	Dataset-Level Heterogeneity/Limitation
Al-Masni et al., 2018 [86]	Full-resolution convolutional network	ISBI 2017, PH2; dermoscopic images	Internal testing on benchmark datasets	ISBI 2017: Jaccard 77.11%, accuracy 94.03%; PH2: Jaccard 84.79%, accuracy 95.08%	NR	Partial cross-dataset reporting using PH2	Dataset size and annotation protocols differ between ISBI and PH2; limited demographic metadata
Vesal et al., 2018 [87]	SkinNet, modified U-Net	ISBI 2017; dermoscopic images	5-fold cross-validation/challenge test setting	Dice 85.10%, Jaccard 76.67%, sensitivity 93.0%	NR	No independent clinical cohort	Good benchmark performance, but limited evidence for device/population generalization
Goyal et al., 2019 [88]	Ensemble Mask R-CNN + DeepLabv3+	ISIC 2017; dermoscopic images	ISIC 2017 train/test setting	Jaccard 79.58%; class-wise accuracy: benign 95.60%, melanoma 90.78%, seborrheic keratosis 91.29%	NR	No	Useful class-level reporting, but no confidence intervals or prospective testing
Sarker et al., 2021 [89]	SLSNet lightweight GAN-based segmentation	ISBI 2017 and ISIC 2018; dermoscopic images	Benchmark-based testing	Accuracy 97.61%, Dice 90.63%, Jaccard 81.98%; >110 FPS on GTX1080Ti	NR	No independent clinical validation	Strong efficiency reporting, but external generalization and fairness not assessed
Innani et al., 2023 [90]	Efficient-GAN/MGAN lesion segmentation	ISIC 2018; dermoscopic images	Internal benchmark evaluation	Dice 90.1%, Jaccard 83.6%, accuracy 94.5%	NR	No	Strong internal segmentation performance; limited evidence under external domain shift

**Table 5:** Comparison of representative skin lesion classification studies.

Study/Link	Model/Architecture	Dataset/Image Type	Validation Design	Reported Metrics	CI/SD/Variance	External Validation	Statistical/Comparative Analysis	Dataset-Level Heterogeneity/Limitation
Esteva et al., 2017 [92]	Deep CNN trained end-to-end	129,450 clinical images; 2032 diseases	Tested against 21 board-certified dermatologists on biopsy-proven images	Performance comparable to dermatologists for keratinocyte carcinoma and melanoma classification	NR in accessible abstract-level reporting	No broad multi-institutional prospective validation	Human-expert comparison included	Large training set, but demographic and device-level heterogeneity incompletely reported
Gouda et al., 2022 [94]	CNN, ResNet50, InceptionV3, Inception-ResNet	ISIC 2018; dermoscopic images	Internal validation	CNN accuracy 83.2%; ResNet50 83.7%; InceptionV3 85.8%; Inception-ResNet 84.0%	NR	No	Architecture comparison included	Internal benchmark performance; limited fairness and external validation
Shetty et al., 2022 [95]	Machine-learning models and CNN	HAM10000; dermoscopic images	10-fold cross-validation	Highest CNN accuracy 95.18%	NR	No	CNN compared with machine-learning classifiers	HAM10000 class imbalance noted; no independent external cohort
Khan and Khan, 2023 [96]	SkinViT transformer model	Three melanoma/non-melanoma datasets	Internal testing across three datasets	Accuracy: 0.9109, 0.8611, 0.8911; AUC: 0.9711, 0.9459, 0.9595	NR	No true external clinical validation	Compared with EfficientNetV2, MaxViT, MobileViTV2, and ViT	Dataset-level differences exist, but prospective validation and skin-tone subgroup analysis not reported
Tschandl et al., 2020 [97]	CNN-based AI support for human–computer collaboration	Dermoscopic images/telemedical setting	Reader study with AI support	Reported improvement in human–AI decision-making; statistical testing used	Statistical testing reported; detailed CI should be extracted from full text	Clinical-reader setting but not full prospective deployment	Two-sided paired t-tests with Holm–Bonferroni correction reported	Strong clinical relevance, but AI performance depends on reader interaction and setting

**Table 6:** Traceability-enhanced summary of generative augmentation and multimodal studies.

Study/Paper Link	Approach	Dataset/Modality	Reported Performance or Impact	CI/SD/Statistical Comparison	Key Heterogeneity/Limitation	Evidence Interpretation
Ding et al., 2020/2021 [103]	Conditional GAN for high-resolution dermoscopy synthesis	Dermoscopy images with segmentation/category conditioning	Image synthesis study; not a direct diagnostic AUC/Dice comparison	NR	Synthetic-image fidelity and downstream clinical benefit need separate validation	Useful for augmentation research, but not sufficient as diagnostic evidence
Kawahara et al., 2019 [104]	Multitask multimodal neural network	Derm7pt; clinical + dermoscopic images + metadata; 1011 lesion cases	Comprehensive diagnostic and seven-point-checklist outputs reported	CI NR in accessible summary	Moderate dataset size; missing metadata can affect reproducibility	Important multimodal reference; metrics should be interpreted task-wise
Lyakhov et al., 2023 [105]	Multimodal neural network with modified cross-entropy loss for imbalance	Heterogeneous dermatological data	Accuracy 85.19%; McNemar statistical significance reported as *p* = 0.001 in comparative tests	Statistical comparison reported	Dataset imbalance addressed, but broader external fairness validation remains needed	Stronger than purely narrative multimodal claims because statistical testing is included
Zhang et al., 2023 [106]	Throughout Fusion Transformer/TFormer	Multimodal skin-lesion diagnosis using dermoscopic/clinical images and metadata	Performance reported in original paper; use source-specific metrics only after extraction	CI NR in accessible summary	Multimodal fusion architecture; computational cost and metadata completeness are key concerns	Useful emerging transformer-based multimodal method; requires careful metric traceability

**Table 7:** Key limitations and future recommendations for skin-lesion AI systems.

Limitation	Current Challenge	Future Recommendation
Dataset bias	Public datasets often show class imbalance, limited rare lesions, and under-representation of darker skin tones	Build multi-source datasets with balanced lesion classes, Fitzpatrick skin type, anatomical site, device, and demographic metadata
Weak benchmark design	Random internal splits may inflate performance	Use patient-level splitting, duplicate removal, fixed test sets, and transparent preprocessing protocols
Limited external validation	Many models are evaluated only on internal test sets	Require cross-dataset, multi-institutional, and prospective validation before clinical claims
Skin-tone fairness	Performance is rarely reported across Fitzpatrick skin types	Report AUC, sensitivity, specificity, false-negative rate, and calibration across Fitzpatrick I–VI
Metric overinterpretation	High AUC, Dice, or accuracy may not indicate clinical reliability	Report confidence intervals, threshold-selection strategy, calibration, and subgroup metrics
Limited interpretability	Heatmaps and saliency maps may highlight artifacts or non-lesion regions	Validate explanations against expert lesion regions and clinically meaningful dermoscopic features
Lack of uncertainty estimation	Models may provide confident outputs for poor-quality or unfamiliar images	Add uncertainty scoring, out-of-distribution detection, and dermatologist-referral triggers
Deployment gap	Most systems are tested offline rather than in real clinical workflows	Conduct workflow studies measuring diagnostic support, clinician trust, usability, safety, and patient outcomes
Privacy and scalability	Centralized data collection may be difficult across hospitals	Explore federated learning, privacy-preserving validation, and secure multi-center model development
Resource constraints	Large models may be unsuitable for mobile or low-resource settings	Use lightweight architectures, compression, quantization, and edge-compatible deployment

## Data Availability

The review data supporting this systematic review are provided as Supplementary Materials.

## Supplementary Materials

The following supporting information can be downloaded at: https://www.mdpi.com/article/10.3390/bioengineering13080872/s1.
