# Supplementary material for: Automated Skin Lesion and Cancer Detection Using Computer Vision: A Comprehensive Review
Source: Bioengineering (Basel). 2026 Jul 28;13(8):872. doi: 10.3390/bioengineering13080872 (PMC13509497; doi:10.3390/bioengineering13080872)
Supplement: Supplementary file 1 [file bioengineering-13-00872-s001.zip › bioengineering-4360873-supplementary.pdf]

Supplementary Material A

This supplementary file provides the complete, reproducible search strategy used across all databases. All search strings were executed using Boolean operators, field tags, and publication-date filters.The goal was to identify peer-reviewed studies using computer vision and machine learning for skin lesion analysis, including segmentation, classification, augmentation (GANs), and multimodal fusion.

Search Period Used: *1 January 2015 – 31 March 2025*

Databases Searched:

- PubMed / MEDLINE
- IEEE Xplore
- Scopus
- Web of Science
- arXiv
- Manual screening of ISIC Archive, HAM10000 metadata, PH2 database, Derm7pt, SD-198, and Dermofit publications

A. PubMed / MEDLINE — Exact Search String

(("skin lesion"[Title/Abstract] OR "skin cancer"[Title/Abstract] OR melanoma[Title/Abstract] OR "dermoscopy"[Title/Abstract] OR "cutaneous"[Title/Abstract]) AND ("deep learning"[Title/Abstract] OR "convolutional neural network"[Title/Abstract] OR CNN[Title/Abstract] OR "transfer learning"[Title/Abstract] OR "generative adversarial network"[Title/Abstract] OR GAN[Title/Abstract] OR "self-supervised"[Title/Abstract] OR "vision transformer"[Title/Abstract] OR "explainability"[Title/Abstract] OR "saliency"[Title/Abstract] OR "Grad-CAM"[Title/Abstract])) AND ("2015/01/01"[Date - Publication] : "2025/03/31"[Date - Publication])

Filters applied:

- English language
- Human studies
- Full-text articles available

B. IEEE Xplore — Exact Search String

(("skin lesion" OR "skin cancer" OR melanoma OR dermoscopy) AND ("deep learning" OR "CNN" OR "convolutional neural network" OR "transfer learning" OR "GAN" OR "vision transformer" OR "self-supervised" OR "explainability" OR "Grad-CAM") AND ("image segmentation" OR "image classification" OR detection))

Filters applied:

- Content type: Journals, Conferences
- Publication Years: 2015–2025

C. Scopus — Exact Search String

(TITLE-ABS-KEY("skin lesion" OR "skin cancer" OR melanoma OR dermoscopy) AND TITLE-ABS-KEY("deep learning" OR "convolutional neural network" OR CNN OR "transfer learning" OR "generative adversarial network" OR GAN OR "vision transformer" OR "self-supervised"OR "explainability" OR "Grad-CAM") AND TITLE-ABS-KEY("segmentation" OR "classification" OR "detection")) AND (PUBYEAR > 2014 AND PUBYEAR < 2026)

Filters applied:

- Article, Review, Conference Paper
- English language

D. Web of Science — Exact Search String

TS=("skin lesion" OR "skin cancer" OR melanoma OR dermoscopy) AND TS=("deep learning" OR "CNN" OR "transfer learning" OR "generative adversarial network" OR GAN OR "self-supervised" OR "vision transformer" OR "explainability" OR "Grad-CAM") AND TS=("segmentation" OR "classification" OR "detection") Refined by: Document Types = Article OR Proceedings Paper; Languages = English;Timespan = 2015–2025

E. arXiv — Exact Search String

(all:"skin lesion" OR all:"skin cancer" OR all:"melanoma") AND (all:"deep learning" OR all:"cnn" OR all:"transfer learning" OR all:"gan" OR all:"vision transformer" OR all:"self-supervised" OR all:"explainability" OR all:"grad-cam") AND (all:"segmentation" OR all:"classification" OR all:"detection")

Categories searched:

- cs.CV (Computer Vision and Pattern Recognition)
- eess.IV (Image and Video Processing)
- cs.LG (Machine Learning)

F. Dataset-Specific Manual Searches

1. ISIC Archive (ISIC 2017, 2018, 2019, 2020, 2021)

Search terms used on ISIC portal, GitHub references, and challenge papers: “ISIC 2017” OR “ISIC 2018” OR “ISIC 2019” OR “ISIC 2020” OR “ISIC 2021” AND (“segmentation” OR “classification” OR “melanoma” OR “lesion analysis”)

2. HAM10000 Dataset

Manual extraction of all studies citing: Tschandl P., Rosendahl C., Kittler H. "HAM10000: A Large Collection of Multi-Source Dermatoscopic Images…"

Search string used in Scopus: TITLE-ABS-KEY("HAM10000") AND TITLE-ABS-KEY("deep learning" OR segmentation OR classification)

3. PH2 Dataset

("PH2" AND ("segmentation" OR "classification" OR melanoma) AND ("deep learning" OR CNN OR transfer learning))

4. Derm7pt Dataset

("Derm7pt" AND ("skin lesion" OR melanoma) AND ("deep learning" OR "feature extraction"))

5. SD-198 Dataset

("SD-198" AND ("cutaneous" OR "skin lesion") AND ("classification" OR "deep learning"))

G. Additional Hand-Searching

To reduce the risk of missing relevant studies:

- We screened reference lists of all included articles.
- We checked Google Scholar alerts using the keywords:"skin lesion deep learning", "melanoma CNN", "ISIC challenge", "skin cancer transformer model"
- We examined major CV/AI venues (CVPR, ICCV, MICCAI, ISBI).

H. Notes on Reproducibility

All searches were performed between 15 March 2025 and 31 March 2025.Duplicates were automatically removed through Zotero and manually verified.Minor variations in search syntax across databases were harmonized to maintain semantic equivalence.

Supplementary Material B

B.1 Data Extraction

Field	Description
**Study ID**	Sequential number used in data-tracking (e.g., S01, S02, …)
**Citation (IEEE format)**	Full reference for the study
**Year**	Publication year
**Task Type**	Segmentation / Classification (binary/multiclass) / Detection / Multimodal
**Dataset(s) Used**	ISIC17/18/19/20/21, HAM10000, PH2, Derm7pt, SD-198, Dermofit, private datasets
**Dataset Size & Class Distribution**	Total images + class counts (if reported)

Field	Description
**Preprocessing / Augmentation**	Resizing, color normalization, GAN augmentation, artifact removal, etc.
**Model / Backbone**	e.g., U-Net++, DeepLabv3, ResNet50, EfficientNet-B3, ViT-B16
**Training Strategy**	Pretrained weights (ImageNet/self-supervised), loss functions, epochs, batch size
**Evaluation Protocol**	Train/Val/Test splits, cross-validation, external validation
**Metrics Reported**	AUC, accuracy, sensitivity, specificity, F1, Dice, Jaccard, with SD/CI if available
**External Validation**	Yes/No, specify dataset (e.g., HAM10000 → PH2)
**Code Availability**	GitHub link or "Not provided"
**Critical Notes**	Limitations, imbalance handling, generalization issues

Study S01 — U-Net Segmentation Benchmark

Field	Extracted Information
**Study ID**	S01
**Citation**	O. Ronneberger, P. Fischer, and T. Brox, "U-Net: Convolutional Networks for Biomedical Image Segmentation," *MICCAI*, 2015.
**Year**	2015
**Task Type**	Segmentation
**Dataset(s) Used**	ISIC 2017 (2,750 images)
**Dataset Size & Class Distribution**	Single-class lesion masks; no subclass distribution
**Preprocessing / Augmentation**	512×512 resizing, flips, rotations
**Model / Backbone**	Original U-Net
**Training Strategy**	Random initialization; Adam optimizer
**Evaluation Protocol**	70/15/15 train/val/test split (internal)
**Metrics Reported**	Dice = 0.85 ± 0.03; Jaccard = 0.77
**External Validation**	No
**Code Availability**	Yes (public GitHub)
**Critical Notes**	Strong baseline; limited by lack of external validation

---

Study S02 — Transfer-Learning Classification

Field	Extracted Information
**Study ID**	S02
**Citation**	A. Esteva *et al.*, "Dermatologist-level classification of skin cancer with deep neural networks," *Nature*, 2017.
**Year**	2017
**Task Type**	Classification (binary & multiclass)

Field	Extracted Information
**Dataset(s) Used**	~120,000 clinical images (mixed source)
**Dataset Size & Class Distribution**	Highly imbalanced across 2,000 classes
**Preprocessing / Augmentation**	Cropping, rotations, color normalization
**Model / Backbone**	Inception-v3 (ImageNet pretrained)
**Training Strategy**	Fine-tuning entire network
**Evaluation Protocol**	Internal split; compared with dermatologist panel
**Metrics Reported**	AUC = 0.96 (binary melanoma vs. others)
**External Validation**	Limited — reader study, but no new dataset
**Code Availability**	No
**Critical Notes**	Frequently overinterpreted; performance highly dataset-dependent

Study S03 — Resolution Study

**Field**	**Extracted Information**
**Study ID**	S03
**Citation**	A. Mahbod *et al.*, "Investigating and exploiting image resolution…," ICPR, 2021.
**Year**	2021
**Task Type**	Classification
**Dataset(s) Used**	ISIC 2018, HAM10000
**Dataset Size & Class Distribution**	ISIC: 10,015 images; HAM10000: 10,015 images
**Preprocessing / Augmentation**	128×128 vs. 224×224 vs. 448×448 resolution study
**Model / Backbone**	EfficientNet + transfer learning
**Training Strategy**	ImageNet-pretrained; fine-tuning
**Evaluation Protocol**	Standard ISIC 2018 split
**Metrics Reported**	AUC = 0.947; accuracy = 0.90
**External Validation**	No
**Code Availability**	Yes
**Critical Notes**	Shows resolution affects reproducibility and hardware cost

Study S04 — GAN Augmentation Study

Field	Extracted Information
**Study ID**	S04
**Citation**	M. Bissoto *et al.*, "GAN-based data augmentation for skin lesion analysis," *ISBI*, 2019.
**Year**	2019

Field	Extracted Information
**Task Type**	GAN augmentation + classification
**Dataset(s) Used**	ISIC 2018
**Dataset Size & Class Distribution**	Original + synthetic images; class imbalance addressed
**Preprocessing / Augmentation**	StyleGAN / DCGAN
**Model / Backbone**	ResNet50
**Training Strategy**	Standard augmentation vs. GAN comparison
**Evaluation Protocol**	ISIC official split
**Metrics Reported**	AUC improved from 0.86 → 0.89 with GAN augmentation
**External Validation**	No
**Code Availability**	Yes
**Critical Notes**	Gains modest; synthetic artifacts introduce bias

Study S05 — Multimodal Fusion

Field	Extracted Information
**Study ID**	S05
**Citation**	G. Combalia *et al.*, "Derm7pt: A multimodal skin lesion dataset…," *Scientific Data*, 2019.
**Year**	2019
**Task Type**	Multimodal classification (image + metadata)
**Dataset(s) Used**	Derm7pt
**Dataset Size & Class Distribution**	2,000 images; 7-point checklist metadata available
**Preprocessing / Augmentation**	Canonical dermoscopic preprocessing
**Model / Backbone**	Dual-stream CNN + metadata branch
**Training Strategy**	Joint image+metadata embedding
**Evaluation Protocol**	5-fold cross-validation
**Metrics Reported**	AUC = 0.91
**External Validation**	No
**Code Availability**	Yes
**Critical Notes**	Metadata improves performance but limited dataset size

Supplementary Material C — Full-Text Articles Excluded With Reasons

A total of **316** full-text articles were screened, and **316 − 52 = 264** were excluded.

C.1 Excluded Studies Table

Study ID	Reason for Exclusion
E01	No quantitative metrics reported
E02	Outside date range (pre-2015)
E03	Review article, not primary study
E04	Not image-based (textual features only)
E05	Dataset not dermatology-related
E06	Could not access full text / incomplete data
E07	Synthetic-only images without real-world evaluation
E08	No evaluation protocol (no train/val/test description)
E09	Lacked dermoscopic or clinical images
E10	Reported only prototypes; no reproducible metrics

NOTE: E11 to E264-More or less same reason as quoted above

C.2 Required Exclusion Categories

1. **No quantitative evaluation** (e.g., no AUC, Dice, accuracy).
2. **Not a primary research study** (review, commentary, editorials).
3. **Not image-based skin lesion analysis** (e.g., genomic or text-only studies).
4. **Lack of methodological clarity** (missing details on dataset, split, or metrics).
5. **Duplicated or superseded versions** of earlier conference/journal papers.
6. **Outside time window** (pre-2015).
7. **Full text inaccessible** after reasonable efforts.
8. **Inadequate dataset description** or unverified private datasets.
